# Crystal structure and Hirshfeld surface analysis of (*E*)-4-({2,2-di­chloro-1-[4-(di­methyl­amino)­phen­yl]ethenyl}diazen­yl)benzo­nitrile

**DOI:** 10.1107/S2056989021009154

**Published:** 2021-09-07

**Authors:** Namiq Q. Shikhaliyev, Zeliha Atioğlu, Mehmet Akkurt, Gulnar T. Suleymanova, Gulnare V. Babayeva, Sixberth Mlowe

**Affiliations:** a Organic Chemistry Department, Baku State University, Z. Khalilov str. 23, AZ 1148 Baku, Azerbaijan; b Department of Aircraft Electrics and Electronics, School of Applied Sciences, Cappadocia University, Mustafapaşa, 50420 Ürgüp, Nevşehir, Turkey; c Department of Physics, Faculty of Sciences, Erciyes University, 38039 Kayseri, Turkey; d University of Dar es Salaam, Dar es Salaam University College of Education, Department of Chemistry, PO Box 2329, Dar es Salaam, Tanzania

**Keywords:** crystal structure, C—H⋯N inter­actions, C—Cl⋯π inter­actions, π–π stacking inter­actions, Hirshfeld surface analysis

## Abstract

In the title compound, C_17_H_14_Cl_2_N_4_, the dihedral angle between the aromatic rings is 50.09 (9)°. The central –N=N– unit shows an *E* configuration. In the crystal, C—H⋯N inter­actions, C—Cl⋯π and π–π stacking inter­actions [centroid-to-centroid distance = 3.7719 (14) Å] link the mol­ecules, forming mol­ecular layers approximately parallel to the (002) plane. Additional weak van der Waals inter­actions between the layers consolidate the three-dimensional packing. Hirshfeld surface analysis indicates that the most important contributions for the crystal packing are from H⋯H (33.6%), N⋯H/ H⋯N (17.2%), Cl⋯H/H⋯Cl (14.1%) and C⋯H/H⋯C (14.1%) contacts.

## Abstract 2

C—H⋯N inter­actions, C—Cl⋯π inter­actions, and π-π stacking inter­actions link mol­ecules in the crystal, forming mol­ecular layers approximately parallel to the (002) plane. The three-dimensional packing is strengthened by additional weak van der Waals inter­actions between the layers.

## Chemical context   

Azo dyes find numerous applications in a diversity of areas, including in mol­ecular recognition, optical data storage, non-linear optics and as mol­ecular switches, anti­microbial agents, colour-changing materials, liquid crystals, dye-sensitized solar cells, mainly because of the ability for *cis-*to-*trans* isomerization and the chromophoric properties of the –N=N– synthon (Maharramov *et al.*, 2018 ▸; Viswanathan *et al.*, 2019 ▸). Not only isomerization, but azo-hydrazone tautomerisim is also an important phenomenon in the coordination chemistry of azo dyes (Mahmoudi *et al.*, 2018*a* ▸,*b* ▸). Modification of azo dyes with functional groups leads to multifunctional ligands, of which the corresponding metal complexes are effective catalysts in oxidation and in C—C coupling reactions (Ma *et al.*, 2020 ▸, 2021 ▸; Mahmudov *et al.*, 2013 ▸; Mizar *et al.*, 2012 ▸). Moreover, the functional properties of azo dyes are dependent on non-covalent bond-donor or -acceptor site(s) attached to the –N=N– synthon (Gurbanov *et al.*, 2020*a* ▸,*b* ▸; Kopylovich *et al.*, 2011 ▸; Mahmudov *et al.*, 2020 ▸; Shixaliyev *et al.*, 2014 ▸). Thus, we have introduced halogen-bond-donor centres to the –N=N– moiety, leading to a new azo dye, (*E*)-4-({2,2-di­chloro-1-[4-(di­methyl­amino)­phen­yl]ethen­yl}diazen­yl)benzo­nitrile, which provides multiple inter­molecular non-covalent inter­actions.

## Structural commentary   

**Figure 1:**
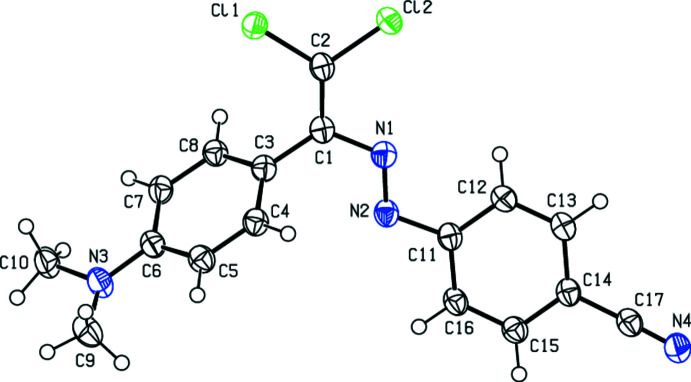
The mol­ecular structure of the title compound, showing the atom labelling and displacement ellipsoids drawn at the 50% probability level.

The aromatic rings C3–C8 and C11–C16 of the title compound (Fig. 1 ▸) form a dihedral angle of 50.09 (9)°. In the di­methyl­amino group, the sum of bond angles about N3 is 357.02° and the nitro­gen atom has a flattened trigonal–pyramidal conformation. The atoms of the di­methyl­amino group and those of its attached benzene ring (C3–C8) are nearly coplanar, with maximum deviations of −0.058 (2), 0.179 (2), and 0.087 (2) Å for N3, C9 and C10, respectively. The title mol­ecule adopts an *E* configuration with respect to the N1=N2 bond. The N1/N2/C1–C3/Cl1/Cl2 unit is approximately planar with a maximum deviation of 0.102 (2) Å, and makes dihedral angles of 55.44 (9) and 5.36 (9)°, respectively, with the C3–C8 and C11–C16 benzene rings.
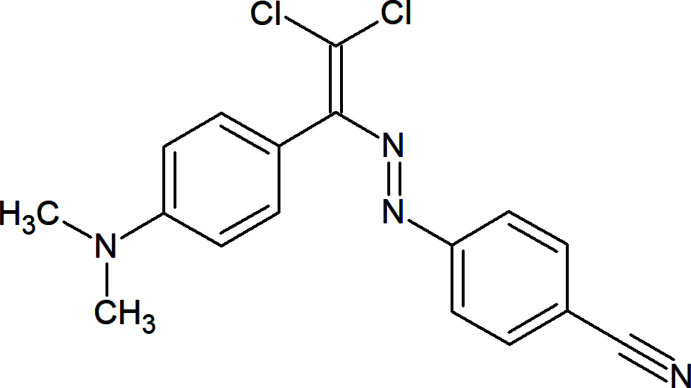


## Supra­molecular features and Hirshfeld surface analysis   

**Figure 2:**
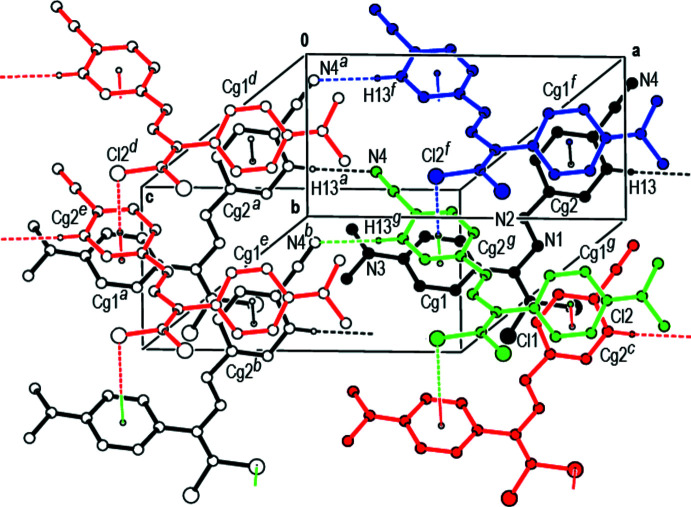
A general view of the C—H⋯N contacts, C—Cl⋯π inter­actions and π–π stacking inter­actions in the crystal packing of the title compound [symmetry codes: (*a*) −1 + *x*, *y*, *z*; (*b*) −1 + *x*, 1 + *y*, *z*; (*c*) *x*, 1 + *y*, *z*; (*d*) 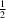 − *x*, −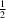 + *y*, 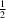 − *z*; (*e*) 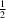 − *x*, 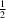 + *y*, 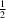 − *z*; (*f*) 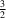 − *x*, −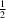 + *y*, 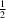 − *z*; (*g*) 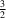 − *x*, 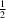 + *y*, 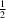 − *z*].

In the crystal, mol­ecules are linked by C—H⋯N inter­actions (Table 1 ▸), C—H⋯π [Cl2⋯*Cg*2^ii^ = 3.3910 (12) Å, C2⋯*Cg*2^ii^ = 3.858 (2) Å, C2—Cl2⋯*Cg*2^ii^ = 92.07 (7)°; symmetry code: (ii) *x*, 1 + *y*, *z*; where *Cg*2 is the centroid of the C11–C16 benzene ring] and π–π stacking inter­actions [*Cg*2⋯*Cg*1^iii^ = 3.7719 (14) Å, slippage = 1.741 Å; *Cg*1⋯*Cg*2^iv^ = 3.7719 (14) Å, slippage = 1.336 Å; symmetry codes: (iii) 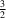 − *x*, − 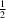 + *y*, 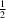 − *z*; (iv) 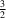 − *x*, 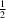 + *y*, 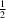 − *z*; where *Cg*1 and *Cg*2 are the centroids of the C3—C8 and C11–C16 benzene rings, respectively], forming mol­ecular layers approximately parallel to the (002) plane with the mol­ecules having a bellows-like shape when viewed along the *a* axis (Figs. 2 ▸ and 3 ▸). Weak van der Waals inter­actions between these layers increase the stability of the crystal structure.

**Figure 4:**
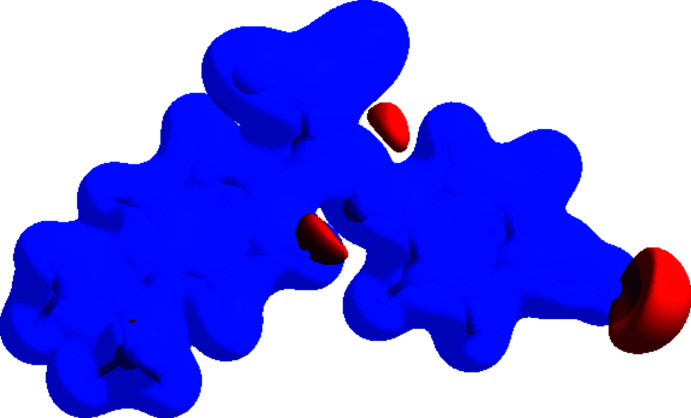
View of the three-dimensional Hirshfeld surface of the title compound plotted over electrostatic potential energy in the range −0.0500 to 0.0500 a.u. using the STO-3 G basis set at the Hartree–Fock level of theory. Hydrogen-bond donors and acceptors are shown as blue and red regions, respectively, around the atoms, corresponding to positive and negative potentials.

**Figure 5:**
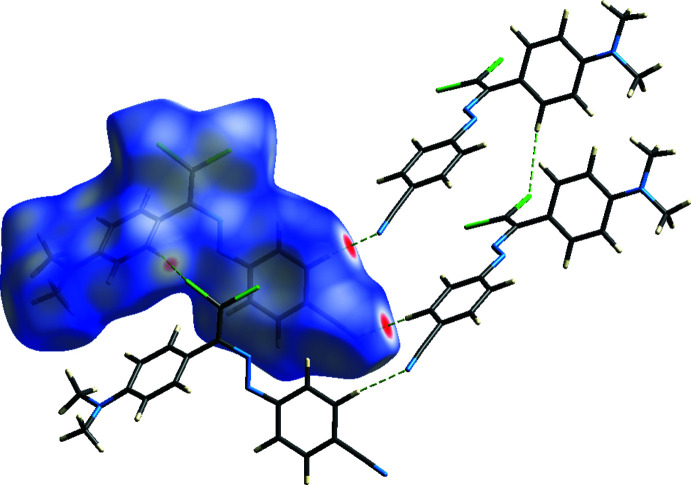
Hirshfeld surface mapped over *d*_norm_ highlighting the regions of C—H⋯Cl and C—H⋯N inter­molecular contacts.

**Figure 6:**
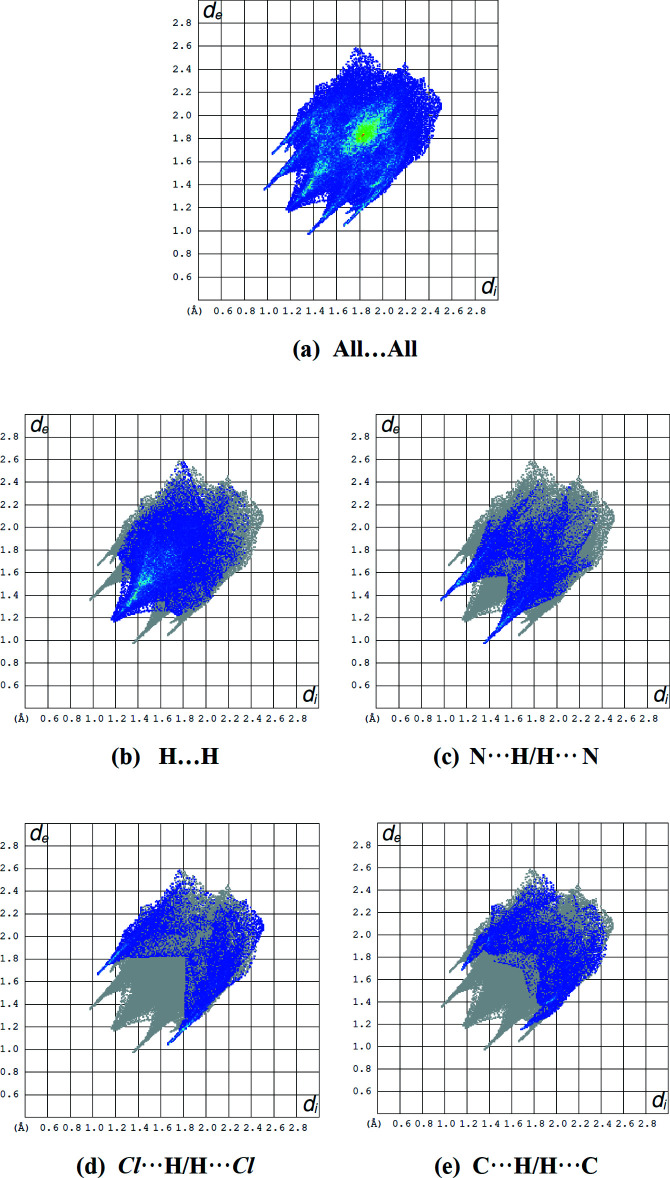
(*a*) The full two-dimensional fingerprint plot for the title compound and those delineated into (*b*) H⋯H (33.6%), (*c*) N⋯H/H⋯N (17.2%), (*d*) Cl⋯H/H⋯Cl (14.1%) and (*e*) C⋯H/H⋯C (14.1%) contacts.

To visualize the inter­molecular inter­actions in the title mol­ecule, *CrystalExplorer17* (Turner *et al.*, 2017 ▸) was used to compute Hirshfeld surfaces (McKinnon *et al.*, 2007 ▸) and their corresponding two-dimensional fingerprint plots (Spackman & McKinnon, 2002 ▸). The Hirshfeld surface mapped over electrostatic potential (Spackman *et al.*, 2008 ▸) is shown in Fig. 4 ▸. The positive electrostatic potential (blue region) over the surface indicates hydrogen-bond donors, whereas the hydrogen-bond acceptors are represented by a negative electrostatic potential (red region). In the Hirshfeld surface mapped over *d*_norm_ (Fig. 5 ▸), the bright-red spots near atoms H7, H13, N4 and Cl1 indicate the short C—H⋯N and C—H⋯Cl contacts (Table 2 ▸). Other contacts are equal to or longer than the sum of van der Waals radii. The most important inter­action is H⋯H, contributing 33.6% to the overall crystal packing, which is reflected in Fig. 6 ▸ *b* as widely scattered points of high density due to the large hydrogen content of the mol­ecule, with the tip at *d*_e_ = *d*_i_ = 1.15 Å. The reciprocal N⋯H/H⋯N inter­actions appear as two symmetrical broad wings with *d*_e_ + *d*_i_ = 2.3 Å and contribute 17.2% to the Hirshfeld surface (Fig. 6 ▸ *c*). The reciprocal Cl⋯H/H⋯Cl inter­actions (14.1% contribution) are present as two symmetrical broad wings with *d*_e_ + *d*_i_ = 2.7 (Fig. 6 ▸ *d*). The pair of characteristic wings in the fingerprint plot delineated into H⋯C/C⋯H contacts (Fig. 6 ▸ *e*; 14.1% contribution) have the tips at *d*_e_ + *d*_i_ = 2.8 Å. The smaller percentage contributions to the Hirshfeld surface from the various other inter­atomic contact are comparatively listed in Table 3 ▸.

## Database survey   

A search of the Cambridge Structural Database (CSD, Version 5.40, update November 2018; Groom *et al.*, 2016 ▸) for structures having an (*E*)-1-(2,2-di­chloro-1-phenylethen­yl)-2-phenyl­diazene unit gave 25 hits. Six compounds closely resemble the title compound, *viz.* 4-{2,2-di­chloro-1-[(*E*)-2-(4-methyl­phen­yl)diazen-1-yl]ethen­yl}-*N*,*N*-di­methyl­aniline [(I); Özkaraca *et al.*, 2020 ▸], 4-{2,2-di­chloro-1-[(*E*)-(4-fluoro­phen­yl)diazen­yl]ethen­yl}-*N*,*N*-di­methyl­aniline [(II); Özkaraca *et al.*, 2020 ▸], 1-(4-chloro­phen­yl)-2-[2,2-di­chloro-1-(4-fluoro­phenyl)ethen­yl]diazene [(III); Shikhaliyev *et al.*, 2019 ▸], 1-(4-bromo­phen­yl)-2-[2,2-di­chloro-1-(4-nitro­phen­yl)ethen­yl]di­azene [(IV); Akkurt *et al.*, 2019 ▸], 1-(4-chloro­phen­yl)-2-[2,2-di­chloro-1-(4-nitro­phen­yl)ethen­yl]diazene [(V); Akkurt *et al.*, 2019 ▸] and 1-[2,2-di­chloro-1-(4-nitro­phen­yl)ethen­yl]-2-(4-fluoro­phen­yl)diazene [(VI); Atioğlu *et al.*, 2019 ▸].

In the crystal of (I), mol­ecules are linked by pairs of C—Cl⋯π inter­actions, forming inversion dimers. A short inter­molecular Cl⋯Cl contact [3.2555 (9) Å] links the dimers, forming a ribbon along the *c*-axis direction. The crystal structure of (II) is stabilized by C—Cl⋯π and van der Waals inter­actions. In (III), mol­ecules are stacked in columns along the *a* axis *via* weak C—H⋯Cl hydrogen bonds and face-to-face π–π stacking inter­actions. The crystal packing is further stabilized by short Cl⋯Cl contacts. In the crystals of (IV) and (V), mol­ecules are linked through weak *X*⋯Cl contacts [*X* = Br for (IV) and Cl for (V)] and C—H⋯Cl and C—Cl⋯π inter­actions into sheets parallel to the *ab* plane. In (VI), mol­ecules are linked by C—H⋯O hydrogen bonds into zigzag chains running along the *c*-axis direction. The crystal packing is further stabilized by C—Cl⋯π, C—F⋯π and N—O⋯π inter­actions.

## Synthesis and crystallization   

The title compound was synthesized according to a reported method (Shikhaliyev *et al.*, 2018 ▸, 2019 ▸). A 20 mL screw-neck vial was charged with DMSO (10 mL), (*Z*)-4-{2-[4-(di­methyl­amino)­benzyl­idene]hydrazin­yl}benzo­nitrile (264 mg, 1 mmol), tetra­methyl­ethylenedi­amine (TMEDA) (295 mg, 2.5 mmol), CuCl (2 mg, 0.02 mmol) and CCl_4_ (20 mmol, 10 equiv). After 1–3 h (until TLC analysis showed complete consumption of the corresponding Schiff base), the reaction mixture was poured into ∼0.01 *M* solution of HCl (100 mL, pH = 2–3), and extracted with di­chloro­methane (3 × 20 mL). The combined organic phase was washed with water (3 × 50 mL) and brine (30 mL), dried over anhydrous Na_2_SO_4_ and concentrated using a vacuum rotary evaporator. The residue was purified by column chromatography on silica gel using appropriate mixtures of hexane and di­chloro­methane (3/1–1/1). Crystals suitable for X-ray analysis were obtained by slow evaporation of an ethanol solution. Colourless solid (69%); m.p. 395 K. Analysis calculated for C_17_H_14_Cl_2_N_4_: C 59.15, H 4.09, N 16.23%; found: C 59.05, H 4.02, N 16.19%. ^1^H NMR (300 MHz, CDCl_3_) *δ* 3.04 (6H, NMe_2_), 6.75–7.89 (8H, Ar). ^13^C NMR (75 MHz, CDCl_3_) δ 162.08, 154.31, 152.59, 146.76, 135.98, 132.50, 131.25, 128.75, 120.90, 117.76, 115.52 and 38.42. ESI–MS: *m*/*z*: 346.18 [*M* + H]^+^.

## Refinement   

Crystal data, data collection and structure refinement details are summarized in Table 4 ▸. The C-bound H atoms were positioned geometrically and treated as riding atoms, C—H = 0.95 Å with *U*_iso_(H) = 1.2*U*_eq_(C) for aromatic H atoms and C—H = 0.98 Å with *U*_iso_(H) = 1.5*U*_eq_(C) for methyl H atoms.

## Supplementary Material

Crystal structure: contains datablock(s) I. DOI: 10.1107/S2056989021009154/vm2253sup1.cif

Structure factors: contains datablock(s) I. DOI: 10.1107/S2056989021009154/vm2253Isup2.hkl

CCDC reference: 2107472

Additional supporting information:  crystallographic information; 3D view; checkCIF report

## Figures and Tables

**Figure 3:**
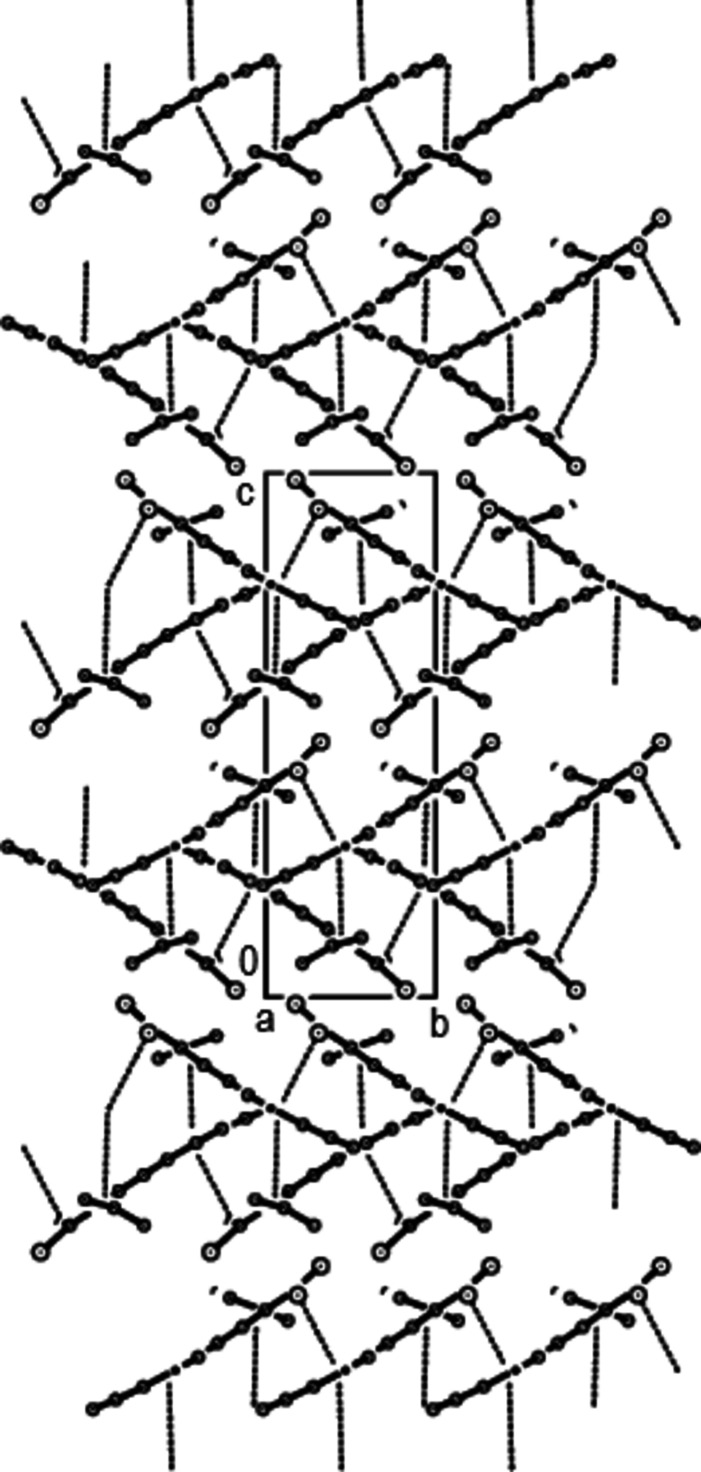
The crystal packing of the title compound, viewed along the *a* axis, showing the C—Cl⋯π inter­actions and π–π stacking inter­actions as dashed lines.

**Table 1:** Hydrogen-bond geometry (Å, °)

*D*—H⋯*A*	*D*—H	H⋯*A*	*D*⋯*A*	*D*—H⋯*A*
C13—H13⋯N4^i^	0.95	2.48	3.428 (3)	175

Symmetry code: (i) -x+{\script{5\over 2}}, y+{\script{1\over 2}}, -z+{\script{1\over 2}}.

**Table 2:** Summary of short inter­atomic contacts (Å) in the title compound

Contact	Distance	Symmetry operation
Cl1⋯H4	2.86	*x*, 1 + *y*, *z*
Cl2⋯Cl1	3.60	2 − *x*, 3 − *y*, 1 − *z*
H9*C*⋯C7	2.95	1 − *x*, 2 − *y*, 1 − *z*
Cl2⋯H10*B*	3.01	1 + *x*, *y*, *z*
C2⋯C2	3.47	2 − *x*, 2 − *y*, 1 − *z*
N4⋯H13	2.48	{5\over 2} − *x*, −{1\over 2} + *y*, {1\over 2} − *z*
N4⋯H7	2.70	{3\over 2} − *x*, −{3\over 2} + *y*, {1\over 2} − *z*

**Table 3:** Percentage contributions of inter­atomic contacts to the Hirshfeld surface for the title compound

Contact	Percentage contribution
H⋯H	33.6
N⋯H/H⋯N	17.2
Cl⋯H/H⋯Cl	14.1
C⋯H/H⋯C	14.1
C⋯C	6.7
Cl⋯C/C⋯Cl	6.3
Cl⋯Cl	3.5
Cl⋯N/N⋯Cl	2.5
N⋯C/C⋯N	1.9
N⋯N	0.1

**Table 4:** Experimental details

Crystal data	
Chemical formula	C_17_H_14_Cl_2_N_4_
*M*_r_	345.22
Crystal system, space group	Monoclinic, *P*2_1_/*n*
Temperature (K)	100
*a*, *b*, *c* (Å)	12.396 (3), 6.5280 (7), 20.758 (3)
β (°)	104.39 (2)
*V* (Å^3^)	1627.1 (5)
*Z*	4
Radiation type	Synchrotron, λ = 0.79475 Å
μ (mm^−1^)	0.54
Crystal size (mm)	0.10 × 0.08 × 0.05

Data collection	
Diffractometer	Rayonix SX165 CCD
Absorption correction	Multi-scan (*SCALA*; Evans, 2006 ▸)
*T*_min_, *T*_max_	0.939, 0.966
No. of measured, independent and observed [*I* > 2σ(*I*)] reflections	21540, 3712, 2913
*R*_int_	0.066
(sin θ/λ)_max_ (Å^−1^)	0.648

Refinement	
*R*[*F*^2^ > 2σ(*F* ^2^)], *wR*(*F* ^2^), *S*	0.040, 0.110, 1.06
No. of reflections	3712
No. of parameters	211
H-atom treatment	H-atom parameters constrained
Δρ_max_, Δρ_min_ (e Å^−3^)	0.34, −0.36

Computer programs: *Marccd*(Doyle, 2011 ▸), *iMosflm* (Battye *et al.*, 2011 ▸), *SHELXT* (Sheldrick, 2015*a* ▸), *SHELXL* (Sheldrick, 2015*b* ▸), *ORTEP-3 for Windows* (Farrugia, 2012 ▸) and *PLATON* (Spek, 2020 ▸).

## supplementary crystallographic information

### Crystal data

**Table:**

C_17_H_14_Cl_2_N_4_	*F*(000) = 712
*M**_r_* = 345.22	*D*_x_ = 1.409 Mg m^−^^3^
Monoclinic, *P*2_1_/*n*	Synchrotron radiation, λ = 0.79475 Å
*a* = 12.396 (3) Å	Cell parameters from 600 reflections
*b* = 6.5280 (7) Å	θ = 2.0–28.0°
*c* = 20.758 (3) Å	µ = 0.54 mm^−^^1^
β = 104.39 (2)°	*T* = 100 K
*V* = 1627.1 (5) Å^3^	Prism, colourless
*Z* = 4	0.10 × 0.08 × 0.05 mm

### Data collection

**Table:**

Rayonix SX165 CCD diffractometer	2913 reflections with *I* > 2σ(*I*)
/f scan	*R*_int_ = 0.066
Absorption correction: multi-scan (*SCALA*; Evans, 2006)	θ_max_ = 31.0°, θ_min_ = 2.0°
*T*_min_ = 0.939, *T*_max_ = 0.966	*h* = −16→16
21540 measured reflections	*k* = −8→8
3712 independent reflections	*l* = −25→26

### Refinement

**Table:**

Refinement on *F*^2^	Secondary atom site location: difference Fourier map
Least-squares matrix: full	Hydrogen site location: inferred from neighbouring sites
*R*[*F*^2^ > 2σ(*F*^2^)] = 0.040	H-atom parameters constrained
*wR*(*F*^2^) = 0.110	*w* = 1/[σ^2^(*F*_o_^2^) + (0.0535*P*)^2^ + 0.5596*P*] where *P* = (*F*_o_^2^ + 2*F*_c_^2^)/3
*S* = 1.06	(Δ/σ)_max_ < 0.001
3712 reflections	Δρ_max_ = 0.34 e Å^−^^3^
211 parameters	Δρ_min_ = −0.36 e Å^−^^3^
0 restraints	Extinction correction: SHELXL, Fc^*^=kFc[1+0.001xFc^2^λ^3^/sin(2θ)]^-1/4^
Primary atom site location: difference Fourier map	Extinction coefficient: 0.0082 (8)

### Special details

**Table:**

Geometry. All esds (except the esd in the dihedral angle between two l.s. planes) are estimated using the full covariance matrix. The cell esds are taken into account individually in the estimation of esds in distances, angles and torsion angles; correlations between esds in cell parameters are only used when they are defined by crystal symmetry. An approximate (isotropic) treatment of cell esds is used for estimating esds involving l.s. planes.

### Fractional atomic coordinates and isotropic or equivalent isotropic displacement parameters (Å^2^)

**Table:**

	*x*	*y*	*z*	*U*_iso_*/*U*_eq_	
Cl1	0.88106 (4)	1.32286 (7)	0.48668 (2)	0.03392 (14)	
Cl2	1.05932 (4)	1.18867 (7)	0.43125 (2)	0.03201 (14)	
N1	0.91524 (13)	0.8624 (3)	0.37007 (8)	0.0292 (3)	
N2	0.85729 (13)	0.7167 (3)	0.33932 (8)	0.0300 (3)	
N3	0.40040 (13)	0.8913 (3)	0.40216 (9)	0.0380 (4)	
N4	1.13398 (14)	−0.0162 (3)	0.21331 (9)	0.0398 (4)	
C1	0.85825 (15)	0.9987 (3)	0.40327 (9)	0.0284 (4)	
C2	0.92392 (15)	1.1508 (3)	0.43572 (9)	0.0295 (4)	
C3	0.73981 (15)	0.9728 (3)	0.40425 (9)	0.0291 (4)	
C4	0.70143 (15)	0.7904 (3)	0.42580 (9)	0.0310 (4)	
H4	0.7525	0.6816	0.4404	0.037*	
C5	0.59082 (16)	0.7638 (3)	0.42649 (10)	0.0330 (4)	
H5	0.5678	0.6384	0.4421	0.040*	
C6	0.51174 (15)	0.9206 (3)	0.40439 (9)	0.0310 (4)	
C7	0.55024 (15)	1.1054 (3)	0.38262 (9)	0.0312 (4)	
H7	0.4994	1.2145	0.3678	0.037*	
C8	0.66197 (15)	1.1295 (3)	0.38267 (9)	0.0294 (4)	
H8	0.6859	1.2551	0.3677	0.035*	
C9	0.36642 (18)	0.7158 (4)	0.43561 (12)	0.0434 (5)	
H9A	0.3835	0.5892	0.4148	0.065*	
H9B	0.2862	0.7231	0.4319	0.065*	
H9C	0.4067	0.7169	0.4826	0.065*	
C10	0.32281 (16)	1.0620 (4)	0.38642 (11)	0.0410 (5)	
H10A	0.3390	1.1609	0.4231	0.061*	
H10B	0.2465	1.0113	0.3800	0.061*	
H10C	0.3307	1.1290	0.3456	0.061*	
C11	0.92081 (15)	0.5780 (3)	0.30993 (9)	0.0291 (4)	
C12	1.03445 (15)	0.6030 (3)	0.31285 (9)	0.0315 (4)	
H12	1.0731	0.7214	0.3330	0.038*	
C13	1.08971 (15)	0.4540 (3)	0.28610 (9)	0.0318 (4)	
H13	1.1666	0.4692	0.2878	0.038*	
C14	1.03148 (15)	0.2803 (3)	0.25645 (9)	0.0297 (4)	
C15	0.91773 (15)	0.2583 (3)	0.25146 (9)	0.0308 (4)	
H15	0.8784	0.1423	0.2299	0.037*	
C16	0.86290 (15)	0.4084 (3)	0.27846 (9)	0.0308 (4)	
H16	0.7855	0.3952	0.2754	0.037*	
C17	1.08914 (15)	0.1179 (3)	0.23155 (10)	0.0329 (4)	

### Atomic displacement parameters (Å^2^)

**Table:**

	*U*^11^	*U*^22^	*U*^33^	*U*^12^	*U*^13^	*U*^23^
Cl1	0.0306 (2)	0.0350 (3)	0.0359 (3)	0.00155 (18)	0.00763 (19)	−0.00490 (19)
Cl2	0.0255 (2)	0.0375 (3)	0.0326 (2)	−0.00150 (18)	0.00657 (17)	0.00029 (19)
N1	0.0276 (7)	0.0326 (9)	0.0261 (8)	0.0018 (6)	0.0045 (6)	0.0004 (6)
N2	0.0254 (7)	0.0358 (9)	0.0275 (8)	0.0029 (6)	0.0043 (6)	−0.0011 (7)
N3	0.0275 (8)	0.0428 (10)	0.0457 (10)	0.0004 (7)	0.0126 (7)	0.0039 (8)
N4	0.0316 (8)	0.0444 (11)	0.0441 (10)	0.0004 (8)	0.0109 (7)	−0.0063 (8)
C1	0.0270 (9)	0.0323 (10)	0.0254 (9)	0.0026 (7)	0.0055 (7)	0.0026 (7)
C2	0.0257 (9)	0.0348 (10)	0.0272 (9)	0.0036 (7)	0.0052 (7)	0.0033 (7)
C3	0.0271 (9)	0.0333 (10)	0.0261 (9)	0.0017 (7)	0.0051 (7)	−0.0007 (7)
C4	0.0284 (9)	0.0334 (11)	0.0300 (9)	0.0034 (7)	0.0049 (7)	0.0010 (8)
C5	0.0313 (9)	0.0375 (11)	0.0304 (10)	−0.0013 (8)	0.0081 (8)	0.0007 (8)
C6	0.0258 (9)	0.0384 (11)	0.0289 (9)	−0.0008 (8)	0.0071 (7)	−0.0017 (8)
C7	0.0276 (9)	0.0352 (10)	0.0301 (9)	0.0048 (8)	0.0062 (7)	−0.0010 (8)
C8	0.0269 (9)	0.0326 (10)	0.0277 (9)	0.0013 (7)	0.0053 (7)	0.0000 (8)
C9	0.0360 (11)	0.0539 (14)	0.0430 (12)	−0.0065 (10)	0.0153 (9)	0.0035 (10)
C10	0.0251 (9)	0.0503 (13)	0.0475 (12)	0.0022 (9)	0.0089 (8)	−0.0047 (10)
C11	0.0258 (9)	0.0345 (11)	0.0264 (9)	0.0030 (7)	0.0052 (7)	0.0024 (8)
C12	0.0266 (9)	0.0347 (10)	0.0326 (10)	−0.0018 (8)	0.0064 (7)	0.0001 (8)
C13	0.0255 (9)	0.0388 (11)	0.0317 (10)	−0.0001 (8)	0.0085 (7)	0.0016 (8)
C14	0.0271 (9)	0.0364 (11)	0.0258 (9)	0.0020 (7)	0.0069 (7)	0.0010 (8)
C15	0.0274 (9)	0.0355 (10)	0.0287 (9)	−0.0008 (8)	0.0057 (7)	0.0000 (8)
C16	0.0231 (8)	0.0403 (11)	0.0283 (9)	−0.0002 (8)	0.0052 (7)	−0.0014 (8)
C17	0.0267 (9)	0.0405 (11)	0.0311 (9)	−0.0009 (8)	0.0064 (7)	−0.0002 (9)

### Geometric parameters (Å, º)

**Table:**

Cl1—C2	1.715 (2)	C7—H7	0.9500
Cl2—C2	1.7217 (19)	C8—H8	0.9500
N1—N2	1.265 (2)	C9—H9A	0.9800
N1—C1	1.417 (2)	C9—H9B	0.9800
N2—C11	1.432 (2)	C9—H9C	0.9800
N3—C6	1.383 (2)	C10—H10A	0.9800
N3—C10	1.456 (3)	C10—H10B	0.9800
N3—C9	1.455 (3)	C10—H10C	0.9800
N4—C17	1.150 (3)	C11—C16	1.391 (3)
C1—C2	1.353 (3)	C11—C12	1.404 (2)
C1—C3	1.483 (2)	C12—C13	1.383 (3)
C3—C4	1.396 (3)	C12—H12	0.9500
C3—C8	1.401 (3)	C13—C14	1.402 (3)
C4—C5	1.386 (3)	C13—H13	0.9500
C4—H4	0.9500	C14—C15	1.395 (3)
C5—C6	1.412 (3)	C14—C17	1.444 (3)
C5—H5	0.9500	C15—C16	1.388 (3)
C6—C7	1.412 (3)	C15—H15	0.9500
C7—C8	1.394 (3)	C16—H16	0.9500
			
N2—N1—C1	115.36 (15)	N3—C9—H9B	109.5
N1—N2—C11	112.77 (15)	H9A—C9—H9B	109.5
C6—N3—C10	120.00 (18)	N3—C9—H9C	109.5
C6—N3—C9	119.85 (18)	H9A—C9—H9C	109.5
C10—N3—C9	117.17 (17)	H9B—C9—H9C	109.5
C2—C1—N1	113.08 (16)	N3—C10—H10A	109.5
C2—C1—C3	123.52 (17)	N3—C10—H10B	109.5
N1—C1—C3	123.36 (17)	H10A—C10—H10B	109.5
C1—C2—Cl1	123.19 (15)	N3—C10—H10C	109.5
C1—C2—Cl2	123.55 (15)	H10A—C10—H10C	109.5
Cl1—C2—Cl2	113.26 (11)	H10B—C10—H10C	109.5
C4—C3—C8	117.53 (17)	C16—C11—C12	120.54 (17)
C4—C3—C1	121.25 (17)	C16—C11—N2	115.41 (16)
C8—C3—C1	121.21 (18)	C12—C11—N2	124.03 (17)
C5—C4—C3	121.80 (18)	C13—C12—C11	119.48 (18)
C5—C4—H4	119.1	C13—C12—H12	120.3
C3—C4—H4	119.1	C11—C12—H12	120.3
C4—C5—C6	120.97 (19)	C12—C13—C14	119.54 (17)
C4—C5—H5	119.5	C12—C13—H13	120.2
C6—C5—H5	119.5	C14—C13—H13	120.2
N3—C6—C5	121.14 (18)	C15—C14—C13	121.10 (18)
N3—C6—C7	121.41 (18)	C15—C14—C17	118.60 (18)
C5—C6—C7	117.41 (17)	C13—C14—C17	120.29 (17)
C8—C7—C6	120.71 (18)	C16—C15—C14	118.99 (18)
C8—C7—H7	119.6	C16—C15—H15	120.5
C6—C7—H7	119.6	C14—C15—H15	120.5
C7—C8—C3	121.57 (19)	C15—C16—C11	120.29 (17)
C7—C8—H8	119.2	C15—C16—H16	119.9
C3—C8—H8	119.2	C11—C16—H16	119.9
N3—C9—H9A	109.5	N4—C17—C14	177.5 (2)
			
C1—N1—N2—C11	−176.74 (15)	C4—C5—C6—C7	−0.9 (3)
N2—N1—C1—C2	179.58 (16)	N3—C6—C7—C8	−177.38 (18)
N2—N1—C1—C3	1.6 (3)	C5—C6—C7—C8	0.5 (3)
N1—C1—C2—Cl1	−173.44 (13)	C6—C7—C8—C3	−0.1 (3)
C3—C1—C2—Cl1	4.5 (3)	C4—C3—C8—C7	0.1 (3)
N1—C1—C2—Cl2	5.4 (2)	C1—C3—C8—C7	179.17 (17)
C3—C1—C2—Cl2	−176.62 (14)	N1—N2—C11—C16	176.66 (16)
C2—C1—C3—C4	−123.2 (2)	N1—N2—C11—C12	−1.9 (3)
N1—C1—C3—C4	54.5 (3)	C16—C11—C12—C13	−2.2 (3)
C2—C1—C3—C8	57.7 (3)	N2—C11—C12—C13	176.31 (17)
N1—C1—C3—C8	−124.5 (2)	C11—C12—C13—C14	0.0 (3)
C8—C3—C4—C5	−0.4 (3)	C12—C13—C14—C15	2.2 (3)
C1—C3—C4—C5	−179.54 (18)	C12—C13—C14—C17	−176.58 (18)
C3—C4—C5—C6	0.9 (3)	C13—C14—C15—C16	−2.2 (3)
C10—N3—C6—C5	173.06 (19)	C17—C14—C15—C16	176.56 (18)
C9—N3—C6—C5	13.1 (3)	C14—C15—C16—C11	0.0 (3)
C10—N3—C6—C7	−9.1 (3)	C12—C11—C16—C15	2.1 (3)
C9—N3—C6—C7	−169.03 (19)	N2—C11—C16—C15	−176.47 (17)
C4—C5—C6—N3	177.02 (18)		

### Hydrogen-bond geometry (Å, º)

**Table:**

*D*—H···*A*	*D*—H	H···*A*	*D*···*A*	*D*—H···*A*
C13—H13···N4^i^	0.95	2.48	3.428 (3)	175

Symmetry code: (i) −*x*+5/2, *y*+1/2, −*z*+1/2.
